# Ten important articles on noninvasive ventilation in critically ill patients and insights for the future: A report of expert opinions

**DOI:** 10.1186/s12871-017-0409-0

**Published:** 2017-09-04

**Authors:** A. Cortegiani, V. Russotto, M. Antonelli, E. Azoulay, A. Carlucci, G. Conti, A. Demoule, M. Ferrer, N.S. Hill, S. Jaber, P. Navalesi, P. Pelosi, R. Scala, C. Gregoretti

**Affiliations:** 1 Department of Biopathology and Medical Biotechnologies (DIBIMED). Section of Anestesia, Analgesia, Intensive Care and Emergency, Policlinico P. Giaccone, University of Palermo, Palermo, Italy; 2 Department of Intensive Care and Anaesthesia, Policlinico A. Gemelli, Catholic University of Rome, Rome, Italy; 3 Réanimation médicale, Hôpital Saint Louis, APHP, Paris, France; 4 Pulmonary Rehabilitation Unit, IRCCS Fondazione S. Maugeri, Pavia, Italy; 5 UMRS1158 Neurophysiologie respiratoire expérimentale et clinique, Sorbonne Universités, UPMC Univ Paris 06, INSERM, Paris, France; 6 AP-HP, Groupe Hospitalier Pitié-Salpêtrière Charles Foix, Service de Pneumologie et Réanimation Médicale (Département “R3S”), 75013 Paris, France; 7 Department of Pneumology, Respiratory Institute, Hospital Clinic-Institut d’Investigacions Biomèdiques August Pi i Sunyer, CibeRes (CB06/06/0028), University of Barcelona, Barcelona, Spain; 8 Division of Pulmonary, Critical Care and Sleep Medicine, Tufts Medical Center, Boston, MA USA; 9 Department of Anesthesiology and Critical Care Medicine B (DAR B), Saint-Eloi Hospital, University Teaching Hospital of Montpellier, Montpellier, France; 10 Anesthesia and Intensive Care, Department of Medical and Surgical Science, Magna Graecia University, Catanzaro, Italy; 11 IRCCS AOU San Martino-IST, Department of Surgical Sciences and Integrated Diagnostics (DISC), IRCCS AOU San Martino IST, University of Genoa, Genoa, Italy; 12 Pulmonology and RICU, S. Donato Hospital, Arezzo, Italy

**Keywords:** Non invasive ventilation, CPAP, Respiratory failure

## Abstract

**Background:**

Noninvasive ventilation is used worldwide in many settings. Its effectiveness has been proven for common clinical conditions in critical care such as cardiogenic pulmonary edema and chronic obstructive pulmonary disease exacerbations.

Since the first pioneering studies of noninvasive ventilation in critical care in the late 1980s, thousands of studies and articles have been published on this topic. Interestingly, some aspects remain controversial (e.g. its use in de-novo hypoxemic respiratory failure, role of sedation, self-induced lung injury). Moreover, the role of NIV has recently been questioned and reconsidered in light of the recent reports of new techniques such as high-flow oxygen nasal therapy.

**Methods:**

We conducted a survey among leading experts on NIV aiming to 1) identify a selection of 10 important articles on NIV in the critical care setting 2) summarize the reasons for the selection of each study 3) offer insights on the future for both clinical application and research on NIV.

**Results:**

The experts selected articles over a span of 26 years, more clustered in the last 15 years. The most voted article studied the role of NIV in acute exacerbation chronic pulmonary disease. Concerning the future of clinical applications for and research on NIV, most of the experts forecast the development of innovative new interfaces more adaptable to patients characteristics, the need for good well-designed large randomized controlled trials of NIV in acute “de novo” hypoxemic respiratory failure (including its comparison with high-flow oxygen nasal therapy) and the development of software-based NIV settings to enhance patient-ventilator synchrony.

**Conclusions:**

The selection made by the experts suggests that some applications of NIV in critical care are supported by solid data (e.g. COPD exacerbation) while others are still waiting for confirmation. Moreover, the identified insights for the future would lead to improved clinical effectiveness, new comparisons and evaluation of its role in still “lack of full evidence” clinical settings.

**Electronic supplementary material:**

The online version of this article (10.1186/s12871-017-0409-0) contains supplementary material, which is available to authorized users.

## Background

Noninvasive ventilation (NIV), as it is commonly known, includes the application of negative pressure to the airways by iron lung or body ventilators, the delivery of noninvasive intermittent positive pressure ventilation (NIPPV) or application of noninvasive continuous positive airway pressure (CPAP) via noninvasive interface. NIV, used as NIPPV or CPAP, is nowadays, a medical intervention used in daily practice worldwide in many clinical settings. Since the first pioneering studies of noninvasive ventilation in critical care in the late 1980s [1–3] thousands of studies and manuscripts have been published on this topic exploring different clinical applications, modalities, interfaces and comparisons with other therapies [4, 5]. Its effectiveness has been proven for common clinical conditions in critical care, such as cardiogenic pulmonary edema (CPE) and exacerbation of chronic obstructive pulmonary disease (COPD) [4–7], as well as for ventilatory support for patients with ventilatory pump failure and to prevent extubation failure [8–10]. However, although extensively studied, some aspects remain controversial such as its role in hypoxemic “de novo” respiratory failure, the best interface and ventilatory modality to optimize patient-ventilator interaction and comfort and side effects (e.g. skin breakdown and non-intentional air-leaks) [11–14]. Recently, its role has been questioned and re-analyzed in light of a widespread use of a new ventilator support mode (high flow nasal therapy - HFNT), better drugs for sedation and emerging patho-physiological concepts such as NIV-induced lung damage due to high transpulmonary pressure/driving pressure or generated tidal volume [15–20]. Thus, during the past decades, some NIV applications have become established, others are being re-examined and need further exploration in the near future. The present survey among some of the worldwide leading experts aims to: 1) select and review 10 important articles published on NIV in critical care setting 2) highlight future directions for both research and clinical applications of this fundamental mode of ventilation that all critical care physicians should be skilled in applying.

## Methods

The selection of experts for this survey by the expert group coordinator (Cesare Gregoretti) and the methodology group was based on: 1) The authors’ rank generated by *Web Of Science* ^*tm*^ for the topic “noninvasive ventilation” under the following categories: “Critical Care Medicine”, “Anesthesiology”, “Emergency Medicine”; 2) recognized scientific impact on this field. The experts received an invitation letter including the aim of the project and the instructions from the experts group coordinator and the methodology group. The experts were asked to select and rank 10 important articles published on NIV, described as both NPPV and CPAP, in critically ill patients. The studies could be randomized controlled trials (RCT), nonrandomized, preclinical, clinical, systematic review or any other type. The experts were also asked not to share their opinion with other experts before finishing the task. Along with the list, the experts gave reasons why they selected each article. After sending their reports, experts received another letter asking them to indicate at least one important insight for research and/or clinical application of NIV for the near future. After the tasks were completed, one investigator from the methodology group assigned a random code to each expert. The investigators who ranked the selections were blind to the experts’ names. The expert group coordinator was asked to provide his list of studies and insights for the future before contacting other experts to avoid potential bias. Sixteen experts were contacted and 12 accepted and completed the task (Massimo Antonelli, Elie Azoulay, Giorgio Conti, Annalisa Carlucci, Alexandre Demoule, Miguel Ferrer, Cesare Gregoretti, Nicholas S. Hill, Samir Jaber, Paolo Navalesi, Paolo Pelosi, Raffaele Scala). Three experts refused and another expert sent his report but the selected studies included not eligible articles and it was eventually excluded. After collecting the forms, the methodology group ranked the studies basing on the number of votes for each study by the experts. In case of *ex aequo*, the methodology group considered for ranking the number of citations from *SCOPUS* database. The insights for the future were also collected and reported.

## Results

The forms completed by the experts are reported in the Additional file 1. Table 1 summarizes the selection of 10 important articles by the experts. We provide the description of the selected studies ranked from tenth to first.

**Table 1:** Ten important articles published on NIV selected by the experts

Rank	Study ID	Title	Votes from the Experts	Citations^a^
1	Brochard L et al. 1995 [34]	Noninvasive ventilation for acute exacerbations of chronic obstructive pulmonary disease	8	1361
2	Nava S et al. 1998 [39]	Noninvasive mechanical ventilation in the weaning of patients with respiratory failure due to chronic obstructive pulmonary disease	8	540
3	Hilbert G et al. 2001 [40]	Noninvasive ventilation in immunosuppressed patients with pulmonary infiltrates, fever, and acute respiratory failure	7	712
4	Antonelli M et al. 1998 [36]	A comparison of noninvasive positive-pressure ventilation and conventional mechanical ventilation in patients with acute respiratory failure	7	670
5	Jaber S et al. 2016 [35]	Effect of noninvasive ventilation on tracheal reintubation among patients with hypoxemic respiratory failure following abdominal surgery. A randomized clinical trial	6	14
6	Plant PK et al. 2000 [33]	Early use of non-invasive ventilation for acute exacerbations of chronic obstructive pulmonary disease on general respiratory wards: a multicenter randomised controlled trial	5	772
7	Brochard L et al. 1990 [3]	Reversal of acute exacerbations of chronic obstructive lung disease by inspiratory assistance with a face mask	5	428
8	Antonelli M et al. 2000 [32]	Noninvasive ventilation for treatment of acute respiratory failure in patients undergoing solid organ transplantation. A randomized trial	4	437
9	Bersten et al. 1991 [1]	Treatment of severe cardiogenic pulmonary edema with continuous positive airway pressure delivered by face mask.	3	431
10	Nava S et al. 2005 [21]	Noninvasive ventilation to prevent respiratory failure after extubation in high-risk patients	3	255

^a^The number of citations for each article was retrieved from SCOPUS at the time of results analysis

### 10. Nava S et al. Crit Care Med. 2005 Nov;33(11):2465–70

Nava et al. [21] in this multi-center RCT enrolled 97 consecutive patients, requiring >48 h of mechanical ventilation and considered at risk of developing post-extubation respiratory failure (i.e., patients who had hypercapnia, congestive heart failure, ineffective cough and excessive tracheobronchial secretions, more than one failure of a weaning trial, more than one comorbid condition, and upper airway obstruction). After a successful weaning trial, patients were extubated and randomized to NIV delivered by a face mask for >8 h/day in the first 48 h (pressure support ventilation – PSV mode, pressure 13 ± 4.5 cmH_2_o and 5.3 ± 1.6 for PEEP) vs standard medical treatment (SMT), namely additional oxygen support. The primary outcome was the need for reintubation according to standardized criteria. Secondary outcomes were intensive care unit (ICU) and hospital mortality, as well as time spent in the intensive care unit and in hospital. Compared with the SMT group, the NIV group had a lower rate of reintubation (4/48 vs. 12/49 - *P* = 0.027) and a lower ICU mortality. The need for reintubation was associated with a higher risk of mortality.

**Why this study?** This is the first randomized controlled study showing a role for NIV as a method for prevention of post-extubation respiratory failure and reintubation in patients at high risk for reintubation. Prior to this study, some not controlled studies demonstrated the efficacy of NIV as after extubation in trauma patients [22] as in unweanable spinal cord injury patients [23]. However, in other prior RCT, NIV had not been found to reduce the need for reintubation in unselected patients with respiratory failure after extubation [24, 25] compared to SMT. Very interestingly, in this study, arterial blood gases were normal at the time of NIV initiation indicating that patients were not in respiratory distress, differently from patients that were enrolled in the former studies on the use of NIV to prevent post-extubation respiratory distress [24, 25]. These findings were also in accordance with data reported by Ferrer et al. published in the same year in a subgroup of COPD patients after a post-hoc analysis [26]. So, we can conclude that NIV should be applied early after extubation without waiting for manifest acute respiratory failure (ARF).

### 9. Bersten AD et al. N Engl J Med. 1991 Dec 26; 325:1825–30

Bersten et al. [1] investigated in a randomized controlled trial whether mask 10 cmH_2_O CPAP delivered via a face mask had physiologic benefit and would reduce the need for intubation and mechanical ventilation in 39 patients with severe CPE and respiratory failure, compared to standard oxygen alone. After 30 min, respiratory rate, pH, arterial carbon dioxide tension and the ratio of Partial Pressure of Arterial Oxygen to the Fraction of Inspired Oxygen (PaO2/FiO2 ratio) had improved significantly more in the CPAP group. Seven out of 20 patients who received oxygen alone but none who received oxygen plus CPAP required intubation and invasive mechanical ventilation (IMV) (*P* = 0.005). At 24 h, no difference was found in respiratory indexes between groups.

**Why this study?** The first randomized trial showing that noninvasive CPAP reduces intubation rate in CPE. One of the earliest reports of positive airway pressure for therapy of CPE was that by Poulton in 1936 [27]. Since then, no controlled study was carried out on this topic. Bersten’s study was able to demonstrate that the main benefits of applying a noninvasive distending pressure, beyond avoiding intubation and improving pulmonary function and oxygenation, are the effects on the heart due to heart-lung interaction. Applying CPAP may lead not only to a decrease in pre-load but also, by decreasing negative pressure swings and, increasing intrathoracic pressure, to a decrease in left-ventricular afterload (namely the transmural ventricular pressure) [28, 29]. This study also led to the publication of other several others studies that tried to demonstrate whether CPAP has any benefit in mortality [30, 31].

### 8. Antonelli M. et al. JAMA. 2000 Jan 12; 283(2):235–41

The study by Antonelli et al. [32] compared face mask NIV (PSV mode to achieve an exhaled tidal volume of 8 to 10 mL/kg with PEEP up to 10 cmH_2_O) vs standard treatment using supplemental oxygen administration to avoid endotracheal intubation in 40 recipients of solid organ transplantation with acute hypoxemic respiratory failure. As previously shown in their former study in immunocompetent hypoxemic patients, within the first hour of treatment significantly more patients in the NIV group improved PaO2/FiO2 ratio. The use of NIV was associated with a significant reduction in the rate of endotracheal intubation (4/20 vs 14/20; *P* = 0.05), rate of fatal complications, length of stay in the ICU by survivors, and ICU mortality.

**Why this study?** The first randomized trial showing potential benefits of NIV for immunocompromised patients with hypoxemic ARF to avoid the potential complications of endotracheal intubation, opening the door for this application of NIV. These results suggested that transplantation programs should consider NIV in the treatment of selected patients with ARF following solid organ transplantation.

### 7. Brochard L et al. N Engl J Med. 1990 Nov 29;323(22):1523–30

Brochard et al. [3] tested the short-term (45-min) physiologic effects of face mask NIV delivered by means of a new dedicated ventilator (PSV mode, pressure 15.5 ± 4,2 cm H_2_O) and a facemask in 11 COPD patients with an acute exacerbation. They found that pH, carbon dioxide, oxygenation and respiratory rate all improved along with a reduction in the diaphragmatic electromyogram signal. In addition, the authors evaluated the potential therapeutic efficacy of NIV in 13 COPD (including 3 of the 11 in physiologic study) who were treated with NIV for several days. Only 1 of these patients needed IMV compared to 11 of the 13 historical controls (*P* < 0.01).

**Why this study?** At the time the study was conducted patients with an acute exacerbations of COPD often required IMV. This seminal study is a “corner stone” in the field of NIV overall for its physiologic findings and for “opening the door” to NIV use for acute exacerbation of COPD patients in the ICU. It demonstrated for the first time the feasibility of using intermittent positive airway pressure delivered by a facemask to obviate the need for IMV in COPD patients with hypercapnic respiratory failure due to an acute exacerbation, the preferred way of managing such patients.

### 6. Plant PK et al. Lancet. 2000 Jun 3;355(9216):1931–5

Plant et al. [33] aimed in a prospective multicentre RCT at finding whether a nasal or face mask NIV use, early after the admission on a general respiratory ward, was effective at reducing the need for IMV and mortality. As done by Brochard et al. in ICU [34] they compared in 236 patients the efficacy of 1) facemask NIV (using bi-level positive airway pressure – PAP – mode) plus SMT vs 2) SMT alone in COPD patients with mild to moderate acidosis. Bi-level positive airway pressure was set was initially set at 10 cm H_2_O of inspiratory pressure and then increased in increments of 5 cmH_2_O to 20 cm H_2_O or the maximum tolerated over 1 h. PEEP was set at 4 cm H_2_O pressure. The major finding of this study was that the early use of NIV for mildly and moderately acidotic patients leads to more rapid improvement of physiological variables, a reduction in the need for invasive mechanical ventilation (with objective criteria), and a reduction in in-hospital mortality.

**Why this study?** At the very beginning of the present century, it was already clear that NIV in an ICU could avert the need for intubation and the mortality associated with severe episodes of COPD exacerbation [3, 34]. However, no study had yet addressed the timing of the use of NIV in a general respiratory ward. This was the first RCT demonstrating the feasibility of using NIV in acute exacerbations of COPD patients outside the ICU. The study supported the idea that early application of NIV in COPD patients with milder ARF improved patients’ outcomes. It furthermore demonstrated that patients with a COPD exacerbation and less severe respiratory acidosis (≥7.30) could be safely managed on a respiratory ward, whilst those with more severe acidosis would need settings with more staff and equipment.

### 5. Jaber S et al. JAMA. 2016 Apr 5;315(13):1345–53

In this multicenter, randomized, parallel-group clinical trial Jaber et al. [35] studied 293 patients admitted to 20 ICU who had undergone abdominal surgery (laparoscopic or nonlaparoscopic elective or nonelective) and developed hypoxemic respiratory failure (PaO2 < 60 mmHg or SpO2 < =90% in association with other signs of respiratory failure). Patients were randomly assigned to receive standard oxygen therapy or face mask NIV (PSV mode, pressure 6.7 ± 3 cm H_2_O and PEEP 5.4 ± 1 cmH_2_O). NIV reduced the risk of tracheal reintubation within 7 days, the primary outcome variable (49/148 vs 66/145 in controls; CI −23.5% to −1.3%; *P* = 0.03). In addition, NIV was associated with significantly more invasive-free days at day 30 and a lower occurrence of health care-associated infections compared with standard oxygen therapy. There were no significant differences in gas exchange or mortality at 90 days.

**Why this study?** The first large positive RCT on the use of NIV for ARF in the post-abdominal surgery that established the role of NIV in reducing the need for invasive mechanical ventilation (IMV). The main finding of the present study was that it was targeted, beyond to find differences in arterial blood gases (secondary outcome), to find differences in tracheal reintubation (primary outcome) for any cause within 7 days of randomization. However mortality did not change at 90 day probably meaning that other factors than invasive mechanical ventilation would interfere with mortality rate.

### 4. Antonelli M et al. New Eng J Med. 1998 Aug 13;339(7):429–35

Antonelli et al. [36] conducted a prospective, randomized trial of face mask NIV (PSV mode with pressure level set above PEEP) vs IMV in 64 patients with hypoxemic ARF requiring mechanical ventilation. PSV was increased to achieve an exhaled tidal volume of 8 to 10 ml per kilogram. PEEP was increased in step of 2 to 3 cm H_2_O up to 10 cm H_2_O.

They showed that NIV was as effective as IMV in improving the PaO2/FiO2 ratio within the first hour (20 of 32 patients – 62% - in the NIV group and 15 of 32–47% - in the IMV group; *P* = 0.21). Ten patients in the NIV group (31%) subsequently required endotracheal intubation. More patients in the IMV group had serious complications (including sinusitis, pneumonia, myocardial infarction, cardiogenic shock, sepsis) and longer periods of ventilation and stays in the ICU.

**Why this study?** Although the role of NIV in hypoxemic patients is nowadays still debated [37] this was the first RCT to suggest a possible role for NIV in patients with hypoxemic ARF. This was also the first and only study comparing intermittent positive NIV (and not only CPAP) with IMV. This study is important because it first proved that NIV could improve arterial blood gases as IMV on the short-term basis.

### 3. Hilbert G et al. N Engl J Med. 2001; Feb 15;344(7):481–7

Hilbert et al. [15] studied 52 immunosuppressed patients (mainly hematologic malignancies) with pneumonitis, fever and ARF comparing intermittent 45 min trial of face mask NIV (PSV mode, pressure 15 ± 2 cm H_2_O and PEEP 6 ± 1 cm H_2_O) plus SMT to SMT alone including supplemental oxygen. The primary outcome was intubation and mechanical ventilation at any time during the study. Significantly fewer patients in the NIV group (12/26–46%) than in the standard-treatment group (20/26–77%) required endotracheal intubation (*P* = 0.03), had serious complications or died in the ICU. Hospital mortality was reduced from 80% in controls to 50% in the NIV group, with 100% mortality among the controls who required intubation.

**Why this study?** This was the first randomized controlled trial showing benefits of using NIV in immunocompromised patients with pulmonary infiltrates, at an early stage of ARF, to prevent complication of IMV. The authors gave the important message that in selected immunosuppressed patients, early NIV initiation can be associated with significant reductions in the use of IMV and its related complications thus improving the likelihood of survival. Very interestingly after many years, avoiding intubation, independently from the NIV use, is still a desirable goal in the management of respiratory failure in immunosuppressed patients to avoid nosocomial infections and bleeding related to hematologic defects [38].

### 2. Nava et al. Ann Inter Med. 1998 May 1;128(9):721–8

In this multicenter, randomized trial Nava et al. [39] compared extubation within 48 h of IMV and application of noninvasive PSV mode (pressure 19.0 ± 2.0 cm H_2_O) through a facial mask compared to invasive PSV in 50 COPD patients admitted in 3 respiratory ICUs. At 60 days, 22 of 25 patients (88%) in the NIV group were successfully weaned compared to 17 of 25 (68%) (*P* < 0.01) who continued with IMV. Early extubation and application of NIV also significantly reduced also the duration of mechanical ventilation, ICU length of stay, and the occurrence of nosocomial pneumonia and was associated with a significantly better 60-day survival rate than IMV.

**Why this study?** At the time of the study, as with the two seminal studies by Brochard et al., IMV was considered the preferred mode of ventilation for COPD patients with ARF [3, 34]. The rate of weaning failure was high leading to prolong mechanical ventilation, predisposing to associated complications. This was the first study showing the usefulness of NIV as a tool for early extubation in selected intubated COPD patients with difficulty weaning. This was also the first study demonstrating that NIV can serve as a technique to avoid re-intubation in this subgroup of patients.

### 1. Brochard L et al. N Engl J Med. 1995 Sep 28;333(13):817–22

Brochard et al. [34] conducted a multicenter, prospective, randomized trial in 85 COPD patients admitted to 5 ICUs with acute exacerbations to compare the efficacy of face mask NIV (PSV mode set initially at 20 cm H2O) plus SMT to SMT alone. Only 11 out of 43 (26%) in the NIV group required intubation, as compared to 31 of 42 (74%) in the standard-treatment group (*P* < 0.001). NIV also significantly reduced the hospital stay, and the in-hospital mortality rate.

**Why this study?** After their study carried out five years before [3] which found that NIV was effective in downloading respiratory muscles and improving arterial blood gases in COPD patients with an acute exacerbations. The authors demonstrated that NIV was able to reduce the proportion of COPD patients with ARF needing IMV and to improve their outcome. It also established for the first time the indication for NIV in COPD exacerbations with respiratory acidosis.

## Discussion

To the best of our knowledge, this is the first report of experts’ selection and opinions on 10 important articles on NIV in critical care. This study also provides future insights on NIV.

The experts selected articles in a span of twenty-six years with the articles more clustered in the last fifteen years. The most voted article was published in 1995 [34]. Nevertheless, one selected article was published in 2016 meaning that NIV is still stimulating high quality research after many years of research of investigation and clinical application [35]. Five selected articles focused on acute exacerbations of COPD inside and outside the ICU, two on NIV use in hypoxemic respiratory failure, two on immunosuppressed and transplant patients and one to prevent respiratory failure after extubation in high-risk patients. This result was expected taking into account the solid evidence for NIV to treat acute exacerbations of COPD. Since the time of the Brochard’s study [34], ranked as 1st, NIV has become the first line therapy when ventilator assistance is deemed indicated in acute exacerbations of COPD.

The study of Hilbert et al. on immunosuppressed patients was ranked as 3rd [40]. Recently, Lemiale et al. found that early NIV compared with standard oxygen therapy alone did not reduce intubation rate or 28-day mortality in immunosuppressed patients with hypoxemic ARF [38]. However, the study has been found to have limitations, including the use of HFNT in nearly 40% of patients in both treatment and control groups [37, 41, 42]. A more recently published subgroup analysis of the outcomes of immunocompromised patients with ARF in the FLORALI (HFNT vs standard oxygen vs NIV) study found higher intubation and mortality rates associated with NIV than HFNT use, further raising concerns about the efficacy of NIV in this subgroup of patients [43]. Indeed, in hypoxemic patients, keep using NIV and thus delaying invasive ventilation may expose the patients to the effects on an increased transpulmonary pressure namely the sum of the pressure applied to the airway by the ventilator and the pleural pressure generated by the patient’s spontaneous effort [41]. The pressure generated by the respiratory muscles added to the level of patient-synchronized pressure support level [44] may generate high tidal volumes higher than those considered safe for lung. In addition, during NIV, the level of positive end expiratory pressure (PEEP) may be not sufficient to recruit consolidated dependent lung areas [45]. Eventually, it may cause a “self induced ventilation lung injury” [46]. However, recently, Demoule et al. found an increase in NIV use and success rate, an overall decrease in mortality, and a decrease of the adverse impact of NIV failure in hypoxemic patients compared to data collected in France 10 years before [47, 48]. This may suggest a link between better patient selection and greater proficiency of staff in administering NIV and patients’ outcome.

Interestingly only one study investigated the use of CPAP in CPE [1]. This is a surprising finding because of the widespread clinical use of CPAP (or PS/PEEP) in CPE [6]. Probably the use of CPAP is so established in many countries that our experts rated only the first pioneering study as important. However, there are still contrasting results between the use of CPAP or NPPV in CPE regarding avoidance of intubation and mortality [30]. The issue is not just whether noninvasive CPAP leads to a decreased mortality, although there is evidence from meta-analysis that it does [6], but also whether it can decrease length of stay, reduce hospital costs and prevent hospital readmissions in heart failure [49].

Absent were some pioneering articles as the one published by Meduri in 1989 [2] and articles related to the use of pre-oxygenation before endotracheal intubation and those referring to patient-ventilator dyssynchrony, use of interfaces as mouth piece [50], or use of NIV in the palliative care setting [51, 52]. Articles on these topics were suggested by some reviewers (see Additional file 1), but were not voted frequently enough by the experts to be ranked in the 10 selected articles [53–55]. Surprisingly there are not studies related to airway humidification during NIV. Humidification and warming of the inspired gas may be needed to prevent the adverse effects of cool, dry gases on the airway epithelium [56]. Data on the level of humidity delivered with different humidification strategies during NIV, demonstrated that active humidifiers and heat and moisture exchangers provide gas with the highest water content compared to no humidification [57].

The experts’ insights and predictions for the future of clinical applications and research for NIV are reported in Table 2. Most of the experts listed one or more of the following points:

1. The need is great for development of innovative new interfaces that adapt better to patient facial characteristics, are more confortable and leak less. The choice of the interface has long been known to be crucial for the success of NIV in both the acute and chronic settings. Type (oral, nasal, nasal pillows, oro-nasal or hybrid mask), size, design, material and headgear may affect the patient’s comfort in many ways, such as discomfort, air-leaks, claustrophobia, skin erythema, eye irritation, skin breakdown and facial deformity in children [58, 59]. However, although the availability of interfaces and the likelihood of meeting patient need is much better today than in the past, for patients needing at least several hours of NIV per day, both in acute and chronic settings, the ideal interface does not yet exist. So the question “facing” the future is: what direction to take to develop better interfaces for NIV?
2. What is the best noninvasive approach to treat hypoxemic respiratory failure? Most of the authors stressed the need for good well-designed, large RCT of NIV in acute “de novo” hypoxemic ARF not restricted to immunosuppressed patients especially to address the role of NIV compared to IMV. In other words, we got the feedback that there is a need for trials to replicate the Frat et al. study [42]. These authors found that the use of HFNT reduced ICU and 90-day mortality as compared to standard oxygen and NIV. The authors speculated that the greater mortality with NIV might have been related to the use of tidal volumes greater than 9 ml/kg, predisposing to ventilator-induced lung injury. However, NIV was used intermittently and not continuously. Moreover, the level of noninvasive pressure-support was only of 8 ± 3 cmH_2_O of water, PEEP only of 5 ± 1 cmH_2_O. In addition it can be hypothesized that the use of other interfaces as helmet [60]. could have increased patient tolerability and time on NIV.
3. Last but not least improving patient-ventilator synchrony still remains an important issue [61]. Developing software-based NIV setting and/or new “fully noninvasive” triggering systems able to enhance patient patient-ventilator synchrony, correct auto-cycling and avoid wasted triggering efforts in presence of moderate or high air leaks [62].

**Table 2:** Future insights for the future of NIV reported by the experts

Expert’s code	What are the insights for the future on NIV?
1	• “A good, well-designed, large randomized controlled trial of NIV vs. oxygen in acute de novo hypoxemic respiratory failure, not specifically designed for immunocompromised patients” • “The perfect interface” • “Reliable early predictors of NIV failure”
2	• “Randomized controlled trial of HFNT + NIV vs continuous NIV vs HFNT in hypoxemic respiratory failure”
3	• “Comparison of HFNT vs NIV in acute hypoxemic respiratory failure, performed according to the usual practice (in the Frat’s paper NIV was not really the clinical practice)”
4	• “The most important insight for the future is the development of HFNT in conjunction and not as an absolute replacement for NIV”
5	• “New inclusion criteria: better definition of patients clinical characteristics to be assisted by NIPPV/CPAP, timing of treatment initiation and interruption” • “New devices: new masks and interfaces more adaptable to patients” • “New comparisons: NIV vs invasive ventilation is hypoxemic respiratory failure and severe COPD. Most of studies have compared NIV with oxygen delivered by a Venturi systems!” • “New modalities of monitoring: development of accurate and precise non invasive CO_2_ monitoring as well as inspiratory efforts during NIV. Development of accurate and precise non invasive monitoring of lung morphological changes during NIV (for example quantitative sonography)”
6	• “Early treatment of acute hypercapnic respiratory failure and hypoxemic respiratory failure, even outside the ICU. • “Mild to moderate hypoxemia in patients who do not improve with HFNT” • “Patients with acute respiratory failure and do-not intubate orders”
7	• “We will see some reduction in use of NIV as HFNT makes inroads into the use of NIV for hypoxemic respiratory failure and perhaps somewhat for hypercapnic respiratory failure (at least in milder cases)” • “Extracorporeal CO_2_ removal will be used instead of NIV in hypercapnic patients at high risk of NIV failure”
8	“The most important insight for the future of NIV will be the development of innovative characteristics of interfaces material, able to improve patient’s comfort and biocompatibility”
10	• “A software-based setting of NIV adjusted according to flow/pressure curves and SpO_2_ to achieve the best PS/PEEP levels and FiO2”
11	• “The most important insight for the future is to succeed in developing a new trigger system able to capture and correct autocycling and wasted effort in a fully noninvasive way, despite the presence of moderate or high air leaks”
12	• “When we evaluate outcomes, we should consider them not only as a result of a binary option of intervention (NIV or no-NIV) but rather as a function of the time spent under each of available interventions (e.g. O2, HFNT, CPAP, IMV) • “We also need to stratify patients according to the underlying pulmonary disease/condition” • “To replicate Antonelli et al. [36] study in other immunocompromized patients population”

*HFNT* high-flow nasal therapy; Expert 9 did not provide the insights

This survey has limitations. Firstly, although the panel included a remarkable number of leading experts, a few did not complete the task after the invitation and others were not invited. It may be possible that, with a higher number of experts we would have had different results. However, we based the selection of experts not only on the rank from a public database and on scientific merit but also on professional background (e.g. intensivists working in general ICU, pulmonologists working in respiratory ICU) in order to include different points of view and to have balanced results. Moreover, 11 out of 12 authors were Europeans. Although it may be argued that a more balanced contribution from experts with cultural diversity would have increased the relevance of the study, no other author from other countries met our inclusion criteria and this may reflect a geographic unbalance in scientific production. Other authors did not give their availability after been asked to participated to the study. Second, the request to the experts was to provide only 10 important studies that globally changed their practice and improve knowledge on the topic globally. Many important studies may have been excluded since ten is a relatively low number considering the very high volume of studies published on this field. Other studies beside on those listed in Table 1 may be retrieved in the Additional file reporting the original lists from the experts.

It may be argued that experts might have been prone to recall and indicate studies in which they had been involved or they were not aware of other articles on the subjects, leading to a bias and potential competing interest. Third, experts’ selection did not include any study with negative results or harmful effects of NIV compared to other forms of ventilatory management [42, 63]. In all areas of knowledge, and mainly in medicine, learning what not to do is so important, or even more, as than the recommendations of what should be done. Although we discussed pathophysiological and clinical insights from these findings, experts’ selection did not include studies with these results.

## Conclusion

We performed an eminence-based selection of 10 important articles published to date on NIV in critical care setting. The articles investigated the use of NIV in COPD exacerbations, in immunocompromized patients, in hypoxemic ARF, in the postoperative management of major abdominal surgical patients and for the treatment of respiratory failure in CPE. Experts identified as insights for future research the development of better interfaces, the use of NIV in the setting of “de-novo” respiratory failure and its comparison with HFNC in a large, well-designed, randomized study and the development of more efficient, software-based, NIV settings to enhance patient-ventilator synchrony.

While some applications of NIV are an important acquisition, after many years from its institution, other are still to be investigated. In conclusion, we are still playing an “ongoing game”.

## Additional file

Additional file 1: Ten important articles on NIV from each expert. Standard forms reporting the articles and the reasons for selection from each expert. The forms are anonymously reported. (PDF 524 kb)

## Abbreviations

ARF: Acute respiratory failure
COPD: Chronic obstructive pulmonary edema
CPAP: Continuous positive airway pressure
CPE: Cardiogenic pulmonary edema
HFNT: High flow nasal therapy
ICU: Intensive Care Unit
IMV: Invasive mechanical ventilation
NIV: Noninvasive ventilation
NPPV: Noninvasive intermittent positive pressure ventilation
PaO2/FiO2: Partial pressure of arterial oxygen to the fraction of inspired oxygen
PAP: Positive airway pressure
PEEP: Positive end expiratory pressure
PSV: Pressure support ventilation
RCT: Randomized controlled trial
SMT: Standard medical treatment
