# Supplementary material for: Ten important articles on noninvasive ventilation in critically ill patients and insights for the future: A report of expert opinions
Source: BMC Anesthesiol. 2017 Sep 4;17:122. doi: 10.1186/s12871-017-0409-0 (PMC5584318; doi:10.1186/s12871-017-0409-0)
Supplement: Additional file 1: — Ten important articles on NIV from each expert. Standard forms reporting the articles and the reasons for selection from each expert. The forms are anonymously reported. (PDF 524 kb) [file 12871_2017_409_MOESM1_ESM.pdf]

The 10 most important articles ever published on NIV. Expert 1	
**Studies**	**Main reasons for selection**
1. Sadoul, Buletin Europen de Physiopathologie Respiratoire, 1965	First demonstration of a physilogical benefit of NIV in acute on chronic respiratory failure
2. Brochard L et al. Noninvasive ventilation for acute exacerbations of chronic obstructive Pulmonary disease. N Engl J Med. 1995 Sep;333(13): 817-22	First RCT on NIV in COPD
3. Masip J et al. Non-invasive pressure support ventilation versus conventional oxygen therapy in acute cargiogenic pulmonary oedema: a randomized trial. Lancet. 2000 Dec 23-30;356(9249):2126-32	Nice RCT on NIV in acute cardiogenic pulmonary edema
4. Demoule et al. Benefits and risks of success or failure of noninvasive ventilation. Intensive Care Med. 2006 Nov;32(11):1756-65	First observation that NIV in hypoxemic respiratory failure may be deleterious
5. Nava et al. Noninvasive ventilation to prevent respiratory failure after extubation in high-risk patients. Crit Care Med. 2005 Nov;33(11):2465-70	First study showing a benefit of Noninvasive ventilation to prevent respiratory failure after extubation in high-risk patients
Hilbert G et al. Noninvasive ventilation in immunosuppressed patients with Pulmonary infiltrates, fever, and acute respiratory failure. N Engl J Med. 2001 Feb 15; 344(7): 481-7	First RCT showing a benefit in immunocompromized patients
7. Antonelli et al. A multiple-center survey on the use in clinical practice of noninvasive ventilation as a first-line intervention for acute respiratory distress syndrome. Crit Care Med. 2007 Jan;35(1):18-25	First observational study describing the potential benefit on NIV in ARDS
8. Fraticelli AT et al. Physiological effects of different interfaces during noninvasive ventilation for acute respiratory failure. Crit Care Med. 2009 Mar;37(3):939-45	A nice comparison of various interfaces
9. Jaber S et al. Effect of Noninvasive Ventilation on Tracheal Reintubation Among Patients With Hypoxemic Respiratory Failure Following Abdominal Surgery: A Randomized Clinical Trial. JAMA. 2016 Apr 5;315(13):1345-53	First large positive RCT on NIV in the post-operative setting
10. Lemiale V et al. Effect of noninvasive ventilation vs oxygen therapy on mortality among immunocompromised patients with acute respiratory failure: a randomized clinical trial. JAMA. 2015 Oct 27;314(16):1711-9	A nice and well done RCT showing that what have been observed 15 year ago is no longer observed

The 10 most important articles ever published on NIV. Expert 2	
**Studies**	**Main reasons for selection**
1. Bersten AD et al. Treatment of severe cardiogenic pulmonary edema with continuous positive airway pressure delivered by face mask.<br>N Engl J Med. 1991 Dec 26;325(26):1825-30	First RCT demonstrated that noninvasive CPAP was able to reduce endotracheal intubation
2. Meduri GU et al. Noninvasive face mask ventilation in patients with acute respiratory failure.<br>Chest. 1989 Apr;95(4):865-70	Seminal study, opening the door of NIV in ICU
3. Brochard L et al. Reversal of acute exacerbations of chronic obstructive lung disease by inspiratory assistance with a face mask.<br>N Engl J Med. 1990 Nov 29;323(22):1523-30	Seminal study on acute exacerbation of COPD patients also describing the physiological application of this technique
4. Antonelli M et al. A comparison of noninvasive positive-pressure ventilation and conventional mechanical ventilation in patients with acute respiratory failure.<br>N Engl J Med. 1998 Aug 13;339(7):429-35	First RCT on hypoxemic ARF and NIV
5. Nava S et al. Noninvasive mechanical ventilation in the weaning of patients with respiratory failure due to chronic obstructive pulmonary disease. A randomized, controlled trial.<br>Ann Intern Med. 1998 May 1;128(9):721-8.	First seminal study on NIV as a tool for weaning COPD patients after an acute exacerbation
6. Calderini E et al. Patient-ventilator asynchrony during noninvasive ventilation: the role of expiratory trigger.<br>Intensive Care Med. 1999 Jul;25(7):662-7	Corner stone study on drawbacks of the PSV use during NIV
7. Plant PK et al. Early use of non-invasive ventilation for acute ex- acerbations of chronic obstructive pulmonary disease on general respiratory wards: a multicentre randomised controlled trial.<br>Lancet 2000 Jun 3;355(9219):1931–5	First RCT demonstrating the feasibility of using NIV in acute exacerbation of COPD patients outside the ICU
8. Carlucci A et al. The configuration of bi-level ventilator circuits may affect compensation for non-intentional leaks during volume-targeted ventilation.<br>Intensive Care Med. 2013 Jan;39(1):59-65	Corner stone study on drawbacks of the use of pressure controlled volume guaranteed mode in presence air leaks
9. Nava S et al. Noninvasive ventilation to prevent respiratory failure after extubation in high-risk patients.<br>Crit Care Med. 2005 Nov;33(11):2465-70	First positive RCT on the use of NIV after extubation in patients at high risk of reintubation
10. Jaber S et al. Effect of Noninvasive Ventilation on Tracheal Reintubation Among Patients With Hypoxemic Respiratory Failure Following Abdominal Surgery: A Randomized Clinical Trial.<br>JAMA. 2016 Apr 5;315(13):1345-53	First study establishing the role of NIV in reducing the need for invasive mechanical ventilation in patients who develop hypoxemic acute respiratory failure after abdominal surgery

The 10 most important articles ever published on NIV. Expert 3	
**Title**	**Main reasons for selection**
1. Taccone P et al. Continuous positive airway pressure delivered with a "helmet": effects on carbon dioxide rebreathing. Crit Care Med. 2004 Oct;32(10):2090-6	No more connected the Helmet-CPAP to a Ventilator in CPAP mode.
2. Vargas F et al. Helmet with specific settings versus facemask for noninvasive ventilation. Crit Care Med. 2009 Jun;37(6):1921-8.	Improving setting to reduce asynchronies due to the high compliance of the helmet
3. Frat JP et al. High-flow oxygen through nasal cannula in acute hypoxemic respiratory failure. N Engl J Med. 2015 Jun 4;372(23):2185-96	Very new option in the early treatment of hypoxemic acute respiratory failure
4. Carlucci A et al. The configuration of bi-level ventilator circuits may affect compensation for non-intentional leaks during volume-targeted ventilation. Intensive Care Med. 2013 Jan;39(1):59-65.	Thinking about the circuit to use when choosing volume-target ventilation
5. Nava S et al. Noninvasive mechanical ventilation in the weaning of patients with respiratory failure due to chronic obstructive pulmonary disease. A randomized, controlled trial. Ann Intern Med. 1998 May 1;128(9):721-8.	Availability of a technique to avoid re-intubation in a subgroup of patients
6. Keenan SP et al. Noninvasive positive-pressure ventilation for postextubation respiratory distress: a randomized controlled trial. JAMA. 2002 Jun 26;287(24):3238-44	Avoiding the progress of acute respiratory failure after extubation where NIV is not superior to medical therapy in avoiding re-intubation
7. L'Her E et al. Physiologic effects of noninvasive ventilation during acute lung injury. Am J Respir Crit Care Med. 2005 Nov 1;172(9):1112-8	Considering to add an inspiratory pressure support in hypoxemic respiratory failure treated by NIV above all if dyspnea or respiratory failure is evident
8. Carteaux G et al. Patient-ventilator asynchrony during noninvasive ventilation: a bench and clinical study. Chest. 2012 Aug;142(2):367-76	Considering the different performance of ventilators in different condition of effort and mechanics, above all considering some NIV ventilators even more performant then ICU ventilators with NIV module.
9. Thille AW et al. Reduction of patient-ventilator asynchrony by reducing tidal volume during pressure-support ventilation. Intensive Care Med. 2008 Aug;34(8):1477-86.	Thinking to reduce firstly PS and secondly Ti when ineffective effort occurs
10. Bach JR et al. Extubation of patients with neuromuscular weakness: a new management paradigm. Chest. 2010 May;137(5):1033-9.	Considering the possibility to use NIV combined to mechanical in-exufflator to wean from invasive ventilation and, in some patients, to significantly reduce the dependence on mechanical ventilation (in term of hours of ventilation)

The 10 most important articles ever published on NIV. Expert 4	
**Studies**	**Main reasons for selection**
1. Brochard L et al. Noninvasive ventilation for acute exacerbations of chronic obstructive Pulmonary disease. N Engl J Med. 1995 Sep;333(13): 817-22.	Established for the first time the indication for NIV in COPD exacerbations with respiratory acidosis.
2. Plant P et al. Early use of non-invasive ventilation for acute exacerbations of chronic obstructive pulmonary disease on general respiratory wards: a multicentre randomised controlled trial. Lancet. 2000 Jun 3; 355(9219): 1931–35.	Demonstrated that patients with COPD exacerbation and less severe respiratory acidosis can be safely managed in wards, whilst those with more severe acidosis would need settings with more staff and equipment.
3. Gray A et al. Noninvasive Ventilation in Acute Cardiogenic Pulmonary Edema. N Engl J Med. 2008 Jul 10; 359(2): 142-51.	The largest clinical trial in acute cardiac failure. Demonstrated that both CPAP and NIV improved equally the management of these patients, but that rapid and aggressive medical treatment was also a key aspect of this
4. Nava S et al. Noninvasive mechanical ventilation in the weaning of patients with respiratory failure due to chronic obstructive pulmonary disease. A randomized controlled trial Ann Intern Med. 1998 May 1;128(9):721-8	Pioneer study on the use of NIV in difficult and prolonged weaning
5. Antonelli M et al. A comparison of noninvasive positive-pressure ventilation and conventional mechanical ventilation in patients with acute respiratory failure N Engl J Med. 1998 Aug 13;339(7):429-35.	Pioneer study on the use of NIV in acute hypoxemic respiratory failure
6. Confalonieri M et al. Acute Respiratory Failure in Patients with Severe Community-acquired Pneumonia. A Prospective Randomized Evaluation of Noninvasive Ventilation. Am J Respir Crit Care Med. 1999 Nov; 160: 1585–1591.	Pioneer study on the use of NIV in community-acquired pneumonia. This study also established that COPD patients and hypercapnic respiratory failure respond better to NIV than those non-COPD without hypercapnia
7. Hilbert G et al. Noninvasive ventilation in immunosuppressed patients with Pulmonary infiltrates, fever, and acute respiratory failure. N Engl J Med. 2001 Feb 15; 344(7): 481-7.	Pioneer study on the use of NIV in immunosuppressed patients with pulmonary infiltrates and acute respiratory failure
8. Ferrer M et al. Early Noninvasive Ventilation Averts Extubation Failure in Patients at Risk. A Randomized Trial. Am J Respir Crit Care Med. 2006 Jan 15: 173(2): 164–170.	Pioneer study on the use of NIV to prevent post-extubation respiratory failure. This study also identified that patients with chronic respiratory disease and hypercapnia during spontaneous breathing were those who benefit better from NIV. This hypothesis was subsequently confirmed in a RCT
9. Girault C et al. Noninvasive Ventilation and Weaning in Patients with Chronic Hypercapnic Respiratory Failure A Randomized Multicenter Trial. Am J Respir Crit Care Med. 2011 Sep 15; 184(6): 672–9.	The largest study on the use of NIV in difficult weaning and in the prevention of post-extubation respiratory failure.
10. Ferrer M et al. Noninvasive Ventilation in Severe Hypoxemic Respiratory Failure. A Randomized Clinical Trial. Am J Respir Crit Care Med 2003 Dec 15;168(12): 1438–44.	This study demonstrated the efficacy of NIV in patients with severe acute hypoxemic respiratory failure, particularly those with pneumonia

The 10 most important articles ever published on NIV. Expert 5	
**Studies**	**Main reasons for selection**
1. Ahlström H et al. Continuous postive airways pressure treatment by a face chamber in idiopathic respiratory distress syndrome.<br>Arch Dis Child. 1976 Jan;51(1):13-21.	As the face chamber is a noninvasive and easily applied technique for CPAP therapy without hazards, it is proposed that it should be used at a still earlier stage of IRDS in order to lesson the need for IPPV treatment and to increase the neurological and lung functional quality of survival. The first study showing potential benefits of non invasive CPAP in infants
2. Meduri GU et al. Noninvasive face mask ventilation in patients with acute respiratory failure.<br>Chest. 1989 Apr;95(4):865-70	First study showing that Noninvasive face mask ventilation may have a role in managing respiratory failure
3. Brochard L et al. Reversal of acute exacerbations of chronic obstructive lung disease by inspiratory assistance with a face mask<br>N Engl J Med. 1990 Nov 29;323(22):1523-30	Inspiratory positive airway pressure delivered by a face mask can obviate the need for conventional mechanical ventilation in patients with acute exacerbations of chronic obstructive pulmonary disease First RCT showing potential benefits of NIV to prevent intubation and improve outcome in acute exacerbation of COPD
4. Bersten AD et al. Treatment of severe cardiogenic pulmonary edema with continuous positive airway pressure delivered by face mask.<br>N Engl J Med. 1991 Dec 26;325(26)1825-1830.	Continuous positive airway pressure delivered by face mask in patients with severe cardiogenic pulmonary edema can result in early physiologic improvement and reduce the need for intubation and mechanical ventilation. This short-term study could not establish whether continuous positive airway pressure has any long-term benefit or whether a larger study would have shown a difference in mortality between the treatment groups.<br>First RCT showing potential benefits of non incvasive CPAP in cardiogenic edema
5. Antonelli M et al. New treatment of acute hypoxemic respiratory failure: noninvasive pressure support ventilation delivered by helmet – a pilot controlled trial<br>Crit Care Med. 2002 Mar;30(3):602-8.	First study showing potential benefits of Helmet to deliver non invasive respiraotry support and avoid intubation
6. Conti G et al. Noninvasive vs. conventional mechanical ventilation in patients with chronic obstructive pulmonary disease after failure of medical treatment in the ward: a randomized trial.<br>Intensive Care Med. 2002 Dec;28(12):1701-7.	The only RCT comparing conventional intubation with non invasive positive pressure ventilation in COPD
7. Antonelli M et al. A comparison of noninvasive positive-pressure ventilation and conventional mechanical ventilation in patients with acute respiratory failure<br>N Engl J Med. 1998 Aug 13;339(7):429-35.	One of the first RCT comparing non invasive positive pressure ventilation and onventional intubation in patients with acute respiratory failure mixed
8. Delclaux C et al. Treatment of acute hypoxemic nonhypercapnic respiratory insufficiency with continuous positive airway pressure delivered by a face mask: A randomized controlled trial.<br>JAMA. 2000 Nov 8;284(18):2352-60.	Study showing no benefits of CPAP in hypoxiemic respiraotry failure

9. Antonelli M et al. Noninvasive ventilation for treatment of acute respiratory failure in patients undergoing solid organ transplantation: a randomized trial.<br>JAMA. 2000 Jan 12;283(2):235-41.	These results indicate that transplantation programs should consider NIV in the treatment of selected recipients of transplantation with acute respiratory failure. RCT showing potential benefits of non invasive ventilation in patients with immunosuppression
10. Frat JP et al. High-flow oxygen through nasal cannula in acute hypoxemic respiratory failure.<br>N Engl J Med. 2015 Jun 4;372(23):2185-96	In patients with nonhypercapnic acute hypoxemic respiratory failure, treatment with high-flow oxygen, standard oxygen, or noninvasive ventilation did not result in significantly different intubation rates. There was a significant difference in favor of high-flow oxygen in 90-day mortality.<br>RCT showing potential benefits of HFNC to prevent intubation and imporve survival in patients with hypoxiemic respiratory failure

The 10 most important articles ever published on NIV. Expert 6	
**Studies**	**Main reasons for selection**
1. Appendini L et al. Physiologic effects of positive end-expiratory pressure and mask pressure support during exacerbations of chronic obstructive pulmonary disease. Am J Respir Crit Care Med. 1994 May;149(5):1069-76.	The best investigation on the mechanisms of NIV in COPD with ARF and on the interplay between PEEP and support
2. Brochard L et al. Noninvasive ventilation for acute exacerbations of chronic obstructive pulmonary disease. N Engl J Med. 1995 Sep 28;333(13):817-22	The study that proved that NIV in COPD with ARF can avoid endotracheal intubation and improve outcome
3. Plant PK et al. Early use of non-invasive ventilation for acute exacerbation of chronic obstructive pulmonary disease on general respiratory wards: a multicenter randomized controlled trial. Lancet. 2000 Jun 3;355(9219):1931-5.	This study indicates that early application of NIV in COPD with ARF improve patient’s outcome
4. Nava S et al. Noninvasive mechanical ventilation in the weaning of patients with respiratory failure due to chronic obstructive pulmonary disease. A randomized controlled trial. Ann Intern Med. 1998 May 1;128:721-728	Seminal on the use of NIV for weaning purposes
5. Nava S et al. Noninvasive ventilation to prevent respiratory failure after extubation in high-risk patients. Crit Care Med 2005 Nov;33(11):2465–2470	The first study showing a role for NIV for prevention of post-extubation respiratory failure and reintubation
6. L’Her E et al. Noninvasive continuous positive pressure in elderly cardiogenic pulmonary edema patients. Intensive Care Med. 2004 May;30(5):882–8	This study first clarified the role and the limitations of CPAP on acute cardiogenic pulmonary edema
7. Antonelli M al. A comparison of noninvasive positive-pressure ventilation and conventional mechanical ventilation in patients with acute respiratory failure. N Engl J Med 1998 Aug 13;339(7):429-35.	Important because first proved that NIV can correct hypoxemia over the short period and may play a role in hypoxemic patients
8. Hilbert G et al. Noninvasive ventilation in immunosuppressed patients with pulmonary infiltrates, fever, and acute respiratory failure. N Engl J Med 2001 Feb 15;344(7):481-7	RCT designing a role for NIV in immunosuppressed patients
9. Calderini E et al. Patient-ventilator asynchrony during noninvasive ventilation: the role of expiratory trigger. Intensive Care Med 1999 Jul;25(7): 662-7	Important because first clarified one of the specific features of NIV, the need for synchronizing patient’s effort and ventilator support with airleaks
10. Schettino GP et al. Mask mechanics and leak dynamics during noninvasive pressure support ventilation: a bench study. Intensive Care Med. 2001 Dec;27(12):1887-91	Underestimated but crucial to understand the interplay between mask tightening, ventilator support and airleaks generation.

The 10 most important articles ever published on NIV. Expert 7	
**Studies**	**Main reasons for selection**
1. Brochard L et al. Noninvasive ventilation for acute exacerbations of chronic obstructive Pulmonary disease. N Engl J Med. 1995 Sep;333(13): 817-22.	More than any other, this article provided solid evidence for the efficacy of NIV to treat acute respiratory failure in patients with COPD.
2. Girou E et al. Secular trends in nosocomial infections and mortality associated with noninvasive ventilation in patients with exacerbation of COPD and pulmonary edema. JAMA. 2003 Dec 10;290(22):2985–91.	This article showed the improved outcomes associated with long-term use of NIV in an ICU to treat acute respiratory failure in COPD and CHF patients – suggesting that accumulated experience and skill of the staff is important in the success of NIV.
3. Esteban A et al. Noninvasive positive-pressure ventilation for respiratory failure after extubation. New Engl J Med. 2004 Jun 10;350(24):2452–60.	This article provided solid epidemiologic evidence for the increasing worldwide use of NIV, especially for COPD patients.
4. Nourdine K et al. Does noninvasive ventilation reduce the ICU nosocomial infection risk? A prospective clinical survey. Intensive Care Med. 1999 Jun;25(6):567–77.	Provided evidence for an important mechanism contributing to improved outcomes of NIV including survival; the reduction in nosocomial infections.
5. Appendini L et al. Physiologic effects of positive end-expiratory pressure and mask pressure support during exacerbations of chronic obstructive pulmonary disease. Am J Respir Crit Care Med. 1994 May;149(5):1069–76.	Elegant study showing the physiologic benefit when applying NIV to COPD patients of combining pressure support plus PEEP as opposed to either one alone.
6. Nava S et al. Noninvasive mechanical ventilation in the weaning of patients with respiratory failure due to chronic obstructive pulmonary disease: A randomized, controlled trial. Ann Intern Med 1998 May 1;128(9):721–8.	First randomized controlled trial (RCT) to demonstrate the value of NIV in facilitating extubation in COPD patients with acute respiratory failure who had required intubation initially.
7. Auriant I et al. Noninvasive ventilation reduces mortality in acute respiratory failure following lung resection. Am J Respir Crit Care Med. 2001 Oct 1; 164(7):1231–5.	First RCT to show reductions in need for intubation and mortality in patients with postoperative respiratory failure following lung resection.
8. Bersten AD et al. Treatment of severe cardiogenic pulmonary edema with continuous positive airway pressure delivered by face mask. N Engl J Med. 1991 Dec 26;325(26)1825-1830.	First randomized trial to show that positive airway pressure (CPAP) reduces intubation rate for treatment of cardiogenic pulmonary edema
9. Hilbert G et al. Noninvasive ventilation in immunosuppressed patients with pulmonary infiltrates, fever, and acute respiratory failure N Engl J Med. 2001 Feb 15;344(7):481-7	First large RCT to demonstrate reductions in intubation and mortality rates in severely immunocompromised patients.
10. Confalonieri M et al. Italian noninvasive positive pressure ventilation study g. A chart of failure risk for noninvasive ventilation in patients with COPD exacerbation. Eur Respir J. 2005 Feb;25(2): 348-55.	First useful chart showing the risk of NIV failure based on a large epidemiologic data base to help in selecting appropriate patients to receive NIV

The 10 most important articles ever published on NIV. Expert 8	
**Studies**	**Main reasons for selection**
1. Brochard L et al. Reversal of acute exacerbations of chronic obstructive lung disease by inspiratory assistance with a face mask. N Engl J Med. 1990 Nov 29;323(22):1523-30	First seminal clinical and physiological study on NIV in acute exacerbation of COPD patients
2. Brochard L et al. Noninvasive ventilation for acute exacerbations of chronic obstructive pulmonary disease. N Engl J Med.1995 Sep 28;333(13):817-22	Since the results of this multicentre RCT, NIV has become the first line treatment in acute exacerbation of COPD patients
3. Antonelli M et al. A comparison of noninvasive positive-pressure ventilation and conventional mechanical ventilation in patients with acute respiratory failure. N Engl J Med. 1998;339:429-35.	First RCT dealing with hypoxemic ARF
4. Antonelli M et al. Noninvasive ventilation for treatment of acute respiratory failure in patients undergoing solid organ transplantation: a randomized trial. Jama. 2000 Jan 12;283(2):235-41	First RCT in immunocompromised patients with hypoxemic ARF
5. Conti G et al. Noninvasive vs. conventional mechanical ventilation in patients with chronic obstructive pulmonary disease after failure of medical treatment in the ward: a randomized trial. Intensive Care Med. 2002 Dec;28(12):1701-7	First RCT showing the feasibility of using NIV as an alternative to invasive ventilation in COPD patients with severe acidosis
6. Plant PK et al. Early use of non-invasive ventilation for acute exacerbations of chronic obstructive pulmonary disease on general respiratory wards: a multicentre randomised controlled trial. Lancet. 2000 Jun 3;355(9219):1931-5	Seminal RCT demonstrating the feasibility of NIV outside the ICU
7. Nava S et al. Noninvasive mechanical ventilation in the weaning of patients with respiratory failure due to chronic obstructive pulmonary disease. A randomized, controlled trial. Ann Intern Med. 199 May 1;128(9):721-8	First RCT study using NIV as a weaning tool in COPD patients after an acute exacerbation
8. L'Her E et al. Physiologic effects of noninvasive ventilation during acute lung injury. Am J Respir Crit Care Med. 2005 Nov 1;172(9):1112-8	Physiologic study investigating the effects of noninvasive intermittent positive pressure ventilation and noninvasive continuous positive airway pressure in patients with hypoxemic ARF
9. Jaber S et al. Effect of Noninvasive Ventilation on Tracheal Reintubation Among Patients With Hypoxemic Respiratory Failure Following Abdominal Surgery: A Randomized Clinical Trial. Jama. 2016 Apr 5;315(5):1345-53	First RCT demonstrating the advantages of NIV in patients with postoperative ARF after abdominal surgery
10. Patel BK et al. Effect of Noninvasive Ventilation Delivered by Helmet vs Face Mask on the Rate of Endotracheal Intubation in Patients With Acute Respiratory Distress Syndrome: A Randomized Clinical Trial. JAMA. 2016 Jun 14;315(22):2435-41	First RCT demonstrating that the delivery of NIV with a helmet was a superior strategy compared to a face mask for ARDS patients

The 10 most important articles ever published on NIV. Expert 9	
**Studies**	**Main reasons for selection**
1. Brochard et al. Reversal of acute exacerbations of chronic obstructive lung disease by inspiratory assistance with a face mask. N Engl J Med. 1990 Nov 29;323(22):1523-30	Main first physiological paper which nicely illustrated the mechanism how NIV could work in COPD patients.
2. Brochard et al. Noninvasive ventilation for acute exacerbations of chronic obstructive pulmonary disease. N Engl J Med. 1995 Sep 28;333(13):817-22	Main first RCT which demonstrated the outcome improvement in COPD patients by NIV.
3. Ferrer et al. Non-invasive ventilation after extubation in hypercapnic patients with chronic respiratory disorders: randomised controlled trial. Lancet. 2009 Sep 26;374(9695):1082-8	First RCT which demonstrated the outcome improvement in selected patients after extubation with "preventive" (or prophylactic) NIV.
4. Gray et al. Noninvasive ventilation in acute cardiogenic pulmonary edema. N Engl J Med. 2008 Jul 10;359(2):142-151	First (large) RCT which evaluated in cardiac patients the application of NIV both CPAP and BIPAP (respectively).
5. Baillard et al. Noninvasive ventilation improves preoxygenation before intubation of hypoxic patients. Am J Respir Crit Care Med. 2006 Jul 15;174(2):171-7	Main first physiological paper which reported that NIV could be used not only to avoid intubation, but it could improve intubation conditions procedure in hypoxemic patients.<br>An innovative indication which is complementary care.
6. Hilbert et al. Noninvasive ventilation in immunosuppressed patients with pulmonary infiltrates, fever, and acute respiratory failure. N Engl J Med. 2001 Feb 15;344(7):481-7	First RCT which demonstrated the outcome improvement in immunosuppressed patients with NIV.
7. Lemiale et al. Effect of Noninvasive Ventilation vs Oxygen Therapy on Mortality Among Immunocompromised Patients With Acute Respiratory Failure: A Randomized Clinical Trial. JAMA. 2015 Oct 27;314(16):1711-9	Second RCT which reported the outcome in immunosuppressed patients with NIV. It is the largest RCT which included the highest number of hypoxemic patients n=374 hypoxemia acute respiratory failure patients.
8. Auriant et al. Noninvasive ventilation reduces mortality in acute respiratory failure following lung resection. Am J Respir Crit Care Med. 2001 Oct 1;164(7):1231-5	First RCT in postoperative area which demonstrated the outcome improvement (decrease the incidence of endotracheal reintubation and mortality) in patients who develop hypoxemia acute respiratory failure after thoracic surgery by application curative nasal NIV.
9. Squadrone et al. Continuous positive airway pressure for treatment of postoperative hypoxemia: a randomized controlled trial. JAMA. 2005 Feb 2;293(5):589-95	Main first RCT which demonstrated the outcome improvement (decrease the incidence of endotracheal intubation and other severe complications) in patients who develop hypoxemia after elective major abdominal surgery by application preventive CPAP.
10. Jaber et al. Effect of Noninvasive Ventilation on Tracheal Reintubation Among Patients With Hypoxemic Respiratory Failure Following Abdominal Surgery: A Randomized Clinical Trial. JAMA. 2016 Apr 5;315(13):1345-53	First large RCT which demonstrated the outcome improvement (decrease the incidence of endotracheal reintubation, nosocomial infections and strong trend decrease in mortality) in patients who develop hypoxemia acute respiratory failure after elective major abdominal surgery by application curative NIV.<br>N=293 hypoxemia acute respiratory failure patients.

The 10 most important articles ever published on NIV. Expert 10	
**Studies**	**Main reasons for selection**
1. Calderini E et al. Patient-ventilator asynchrony during noninvasive ventilation: the role of the expiratory trigger. Intensive Care Med. 1999 Jul;25(7):662-7.	The first study who highlight the effects of leak and setting on patient-ventilator synchrony
2. Carlucci A et al. Changes in the practice of non-invasive ventilation in treating COPD patients over 8 years. Intensive Care Med. 2003 Mar;29(3):419.25	The first real life study who demonstrated the importance of expertise as key ingredient for success of NIV
3. Nava S et al. Noninvasive mechanical ventilation in the weaning of patients with respiratory failure due to chronic obstructive pulmonary disease. A randomized controlled trial. Ann Intern Med. 1998 May 1;128(9):721-8	The first study who showed the usefulness of NIV as tool for early extubation in selected COPD intubated patients with difficult weaning
4. Ferrer M et al. Non-invasive ventilation after extubation in hypercapnic patients with chronic respiratory disorders: randomised controlled trial. Lancet. 2009 Sep 26;374(9695):1082-8	The most convincent study demonstrating the applicability and usefulness of NIV in the management of high risk patients with hypercapnia after extubation by means of NIV
5. Antonelli M et al. Noninvasive positive-pressure ventilation vs. conventional oxygen supplementation in patients undergoing diagnostic bronchoscopy. Chest. 2002 Apr;121(4):1149-54	The first study showing the effectiveness of NIV to support diagnostic bronchoscopy in high risk patients as compared to conventional oxygen therapy
6. Olivieri C et al. Bench comparative evaluation of new generation and standard helmet for delivering non-invasive ventilation Intensive Care Med. 2013 Apr;39(4):734-8	This study depicted the adavntages of the new helmet as compared to standard in terms of better patient-ventilator synchrony
7. Nava S et al. Palliative use of non-invasive ventilation in end-of-life patients with solid tumours: a randomized feasibility trial. Lancet Oncol. 2013 Mar;14(3):219-27	The first RCT showing the feasibility of NIV as palliative care in advanced solid tumors patients with ARF
8. Scala R et al. Noninvasive positive pressure ventilation in patients with acute exacerbations of COPD and varying levels of consciousness. Chest. 2005 Sep;128(3):1657-66	The first study investigating the impact of different levels of hypercapnic encephalopathy on NIV success
9. Hilbert G et al. Noninvasive ventilation in immunosuppressed patients with pulmonary infiltrates, fever, and acute respiratory failure. N Engl J Med. 2001 Feb 15;344(7):481-7	One of the first RCT clearly supporting the use of NIV in immunosuppressed patients for the prevention of intubation-related complications
10. Demoule A et al. Benefits and risks of success or failure of noninvasive ventilation. Intensive Care Med. 2006 Nov;32(11):1756-65	This is a real life experience that strongly underlined the benefit of NIV in hypercapnic ARF an the risks in de novo hypoxemix patients

The 10 most important articles ever published on NIV. Expert 11	
**Studies**	**Main reasons for selection**
1. Brochard L et al. Reversal of acute exacerbations of chronic obstructive lung disease by inspiratory assistance with a face mask. N Engl J Med. 1990 Nov 29;323(22):1523-30	Seminal study, opening the door of NIV in ICU and describing NIV physiology
2. Brochard L et al. Noninvasive ventilation for acute exacerbations of chronic obstructive pulmonary disease. N Engl J Med. 1995 Sep 28;333(13):817-22.	First large multicenter RCT being the basis for NIV as first line treatment in COPD
3. Antonelli M et al. A comparison of noninvasive positive-pressure ventilation and conventional mechanical ventilation in patients with acute respiratory failure. N Engl J Med. 1998 Aug 13;339(7):429-35.	First RCT opening the era of NIV in patients with hypoxemic ARF
4. Antonelli M et al. Noninvasive ventilation for treatment of acute respiratory failure in patients undergoing solid organ transplantation: a randomized trial. JAMA. 2000 Jan 12;283(2):235-41.	First RCT opening the era of NIV in immunocompromised patients with hypoxemic ARF
5. Conti G et al. Noninvasive vs. conventional mechanical ventilation in patients with chronic obstructive pulmonary disease after failure of medical treatment in the ward: a randomized trial. Intensive Care Med. 2002 Dec;28(12):1701-7.	RCT that first showed the applicability of NIV also in COPD patients with severe acidosis as an alternative to invasive ventilation
6. Plant PK et al. Early use of non-invasive ventilation for acute exacerbations of chronic obstructive pulmonary disease on general respiratory wards: a multicentre randomised controlled trial Lancet. 2000 Jun 3;355(9219):1931-5.	Seminal study, opening the door to NIV use outside the ICU
7. Nava S et al. Noninvasive mechanical ventilation in the weaning of patients with respiratory failure due to chronic obstructive pulmonary disease. A randomized, controlled trial. Ann Intern Med. 1998 May 1;128(9):721-8.	First study, describing the advantages of NIV as a weaning tool in pts with COPD exacerbation
8. L'Her E et al. Physiologic effects of noninvasive ventilation during acute lung injury. Am J Respir Crit Care Med. 2005 Nov 1;172(9):1112-8.	Seminal study describing NIV and CPAP physiology in patients with hypoxemic ARF
9. Jaber S et al. Effect of Noninvasive Ventilation on Tracheal Reintubation Among Patients With Hypoxemic Respiratory Failure Following Abdominal Surgery: A Randomized Clinical Trial. JAMA. 2016 Apr 5;315(13):1345-53	First study, describing the advantages of NIV in patients developing postop ARF after abdominal surgery
10. Ferrer M et al. Noninvasive ventilation in severe hypoxemic respiratory failure: a randomized clinical trial. Am J Respir Crit Care Med. 2003 Dec 15;168(12):1438-44.	A large multicentre study describing the advantages of NIV in patients with hypoxemic ARF including pneumonia

The 10 most important articles ever published on NIV. Expert 12	
**Studies**	**Main reasons for selection**
1. Antonelli et al. A comparison of noninvasive positive-pressure ventilation and conventional mechanical ventilation in patients with acute respiratory failure. N Engl J Med 1998 Aug 13 13;339(7):429-35	This is the only RCT that addresses a real clinical question with almost no bias. Should be done again today
2. Jaber et al. Effect of Noninvasive Ventilation on Tracheal Reintubation Among Patients With Hypoxemic Respiratory Failure Following Abdominal Surgery: A Randomized Clinical Trial. JAMA. 2016 Apr 5;315(13):1345-53	A pure example of how the critical literature can be applied outside the ICU with translation into improved outcomes
3. Brochard et al. Noninvasive ventilation for acute exacerbations of chronic obstructive pulmonary disease. N Engl J Med. 1995 Sep 28;333(13):817-22	Landmark paper that has changed the way we deal with COPD in and outside the ICU
4. Lemiale et al. Effect of Noninvasive Ventilation vs Oxygen Therapy on Mortality Among Immunocompromised Patients With Acute Respiratory Failure: A Randomized Clinical Trial. JAMA. 2015 Oct 27;314(16):1711-9	It has challenged a proof of concept paper
5. Hilbert et al. Noninvasive ventilation in immunosuppressed patients with pulmonary infiltrates, fever, and acute respiratory failure. N Engl J Med. 2001 Feb 15;344(7):481-7	This paper – besides IMV – has taken cancer patients back to the ICU
6. Nava S, et al. Palliative use of non-invasive ventilation in end-of-life patients with solid tumours: a randomised feasibility trial. Lancet Oncol. 2013;14(3):219-27	Qualitative outcomes and challenging indication for NIV
7. Antonelli et al. Noninvasive ventilation for treatment of acute respiratory failure in patients undergoing solid organ transplantation: a randomized trial. JAMA. 2000 Jan 12;283(2):235-41	Well done and clean RCT
8. Lorut C et al. Early postoperative prophylactic noninvasive ventilation after major lung resection in COPD patients: a randomized controlled trial. Intensive Care Med. 2014 Feb;40(2):220-7	Has showed that non-ventilation treatments make a difference for use and outcomes associated with NIV. Here peridural analgesia
9. Delclaux et al. Treatment of acute hypoxemic nonhypercapnic respiratory insufficiency with continuous positive airway pressure delivered by a face mask: A randomized controlled trial. JAMA. 2000 Nov 8;284(18):2352-60	The last available paper on CPAP in the ICU outside heart failure
10. Olivieri et al. New versus Conventional Helmet for Delivering Noninvasive Ventilation: A Physiologic, Crossover Randomized Study in Critically Ill Patients. Anesthesiology. 2016 Jan;124(1):101-8	I like this one
